# Overexpression of the *GmDREB6* gene enhances proline accumulation and salt tolerance in genetically modified soybean plants

**DOI:** 10.1038/s41598-019-55895-0

**Published:** 2019-12-23

**Authors:** Quan Huu Nguyen, Lien Thi Kim Vu, Lan Thi Ngoc Nguyen, Nhan Thi Thanh Pham, Yen Thi Hai Nguyen, Son Van Le, Mau Hoang Chu

**Affiliations:** 1 Thainguyen University of Education, Thai Nguyen University, Thai Nguyen, 250000 Viet Nam; 2 Institute of Theoretical and Applied Research, Duy Tan University, Ha Noi, 100000 Viet Nam; 3 Thainguyen University of Sciences, Thai Nguyen University, Thai Nguyen, 250000 Viet Nam; 4 Institute of Biotechnology, Vietnam Academy of Science and Technology, Hanoi, 100000 Vietnam

**Keywords:** Biotechnology, Molecular engineering in plants

## Abstract

Soybean plants are sensitive to the effects of abiotic stress and belong to the group of crops that are less drought and salt tolerant. The identification of genes involved in mechanisms targeted to cope with water shortage is an essential and indispensable task for improving the drought and salt tolerance of soybean. One of the approaches for obtaining lines with increased tolerance is genetic modification. The dehydration-responsive element binding proteins (DREBs), belonging to the AP2 family, are trans-active transcription factors that bind to the cis-sequences of the promoter for activating the expression of the target genes that mediate drought and salt tolerant responses. In this study, the *GmDREB6* transgene was introduced into DT84 cultivar soybean plants, using *Agrobacterium*-mediated transformation. The efficacy of *GmDREB6* overexpression in enhancing the transcriptional level of *GmP5CS* and proline accumulation in genetically modified (GM) soybean plants was also assayed. The results demonstrated that ten GM soybean plants (T0 generation) were successfully generated from the transformed explants after selecting with kanamycin. Among these plantlets, the presence of the *GmDREB6* transgene was confirmed in nine plants by Polymerase Chain Reaction (PCR), and eight plants showed positive results in Southern blot. In the T1 generation, four GM lines, labelled T1-2, T1-4, T1-7, and T1-10, expressed the recombinant GmDREB6 protein. In the T2 generation, the transcriptional levels of the *GmP5CS* gene were higher in the GM lines than in the non-transgenic plants, under normal conditions and also under conditions of salt stress and drought, ranging from 1.36 to 2.01 folds and 1.58 to 3.16 folds that of the non-transgenic plants, respectively. The proline content was higher in the four GM soybean lines, T2-2, T2-4, T2-7, and T2-10 than in the non-transgenic plants, ranging from 0.82 μmol/g to 4.03 μmol/g. The proline content was the highest in the GM T2-7 line (7.77 μmol/g). In GM soybean lines, T2-2, T2-4, T2-7, and T2-10 proline content increased after plants were subjected to salt stress for seven days, in comparison to that under normal conditions, and ranged from 247.83% to 300%, while that of the non-GM plants was 238.22%. These results suggested that *GmDREB6* could act as a potential candidate for genetic engineering for improving tolerance to salt stresses.

## Introduction

Soybean is an important crop with numerous uses and is a source of protein and vegetable oil for human consumption as well as for the animal food industry. Soybean cultivation increases the economic value, nutritive value and fertility of the soil. However, like other crops, soybean plants are exposed to environmental conditions and face the effects of abiotic stresses, including drought and excessive salinity. Drought stress and prolonged drought has significant impacts on the growth and development of soybean plants, reducing the yield and seed quality by approximately 40%, especially if the drought occurs during the stage of grain formation. The resilience of soybean crops against drought and salt stresses is considered to be an adaptation to climate change^1,2^. In order to minimise the negative effects of drought and salt stresses on soybean plants, alternatives such as irrigation systems and strategies for improving soil moisture retention can be employed, in addition to using genetically modified (GM) lines that are more tolerant to these stress conditions. Furthermore, drought and salt tolerant cultivars of soybean are highly sought after, since areas with drought and high salinity are predicted to rise in the future as a result of climate change^3^. Both drought and salt stresses lead to cellular dehydration, which causes osmotic stress and removal of water from the cytoplasm, and consequently a reduction of the cytosolic and vacuolar volumes^4^.

Genetic engineering is a powerful tool for the development of drought and salt tolerant crops, since it allows the modification of gene expression in response to drought and salt stresses. In this context, the identification of genes that are involved in the mechanisms targeted to cope with water shortage and high salinity are essential for the development of GM drought and salt tolerant soybean plants. The advances in understanding gene expression, transcription mechanisms and signal transmission in response to drought have been previously studied^5^. Among the genes that are involved in drought-responses, the genes encoding transcription factors (TFs) are desirable candidates for the genetic engineering of plants, since TFs recognise specific DNA sequences in the promoter region of the target genes and regulate their expression. Acting this way, TFs regulate the expression of several downstream drought and salt stress responsive genes. Some studies have evaluated the role of TFs in improving the drought and salt tolerance of GM soybean plants. DREBs (dehydration responsive element binding proteins) are TFs that are transcriptionally upregulated by water deficiency. The DREBs in soybean, belonging to the AP2 family, are trans-active TFs that bind to cis sequences in the promoter region of the target genes, thus activating their expression in response to abiotic stress signals from the external environment^6,7^. The AP2 domain comprises nearly 58 or 59 amino acids, and consists of certain amino acids that are linked to the dehydration response factor (DRE) or GCC box^8^.

Some studies have demonstrated that plants containing *DREBs* genes have increased tolerance to abiotic stresses in the greenhouse and in the field conditions. These studies also revealed that the yield of GM plants remain stable under conditions of drought, owing to their better drought tolerance response^9^. The AtDREB1A, AtDREB2CA and AtAREB GM soybean lines were analysed in a field under irrigated and non-irrigated conditions. The study confirmed that the drought responses of the GM plants were higher during conditions of drought in the field experiment^10^. These studies concentrated *DREB1* and *DREB2* genes and on yield components, agronomical traits, and physiological parameters. For *GmDREB6* gene no study on the improvement of drought and salt tolerance were performed as well as proline content assayed in GM soybean plants overexpressing *GmDREB6*. Proline is a common osmolyte that increases in plants under conditions of drought, and the accumulation of it increases the osmotic pressure, thus improving plants salt tolerance. Zhang *et al*.^11^ studied the expression of *OsDREB2A* and additionally demonstrated that the expression of this gene increased *DREB6* and *P5CS* genes expression in soybean^11^. However, no data on how the overexpression of soybean *DREB6* increases the expression of *P5CS*, or proline content was assayed in this study.

In this context, our study aimed to analyse the expression of the *GmDREB6* transgene and the efficacy of *GmDREB6* overexpression in enhancing the transcription levels of the *GmP5CS* gene and proline accumulation in GM soybean plants under high salinity conditions.

## Results

### Identification of *35S-GmDREB6-c-myc* construct-positive GM plants

**Figure 1:**
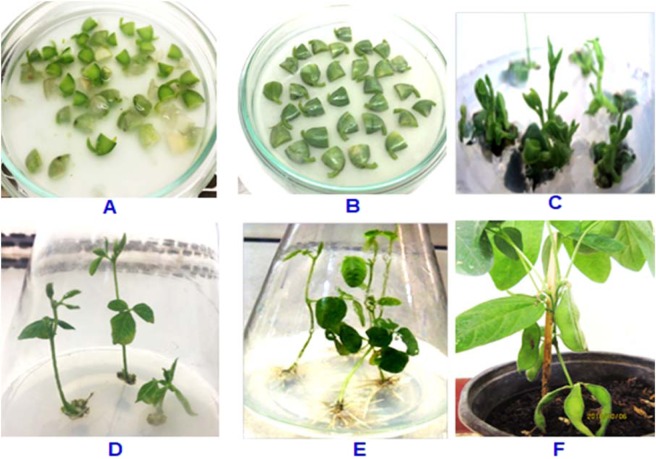
Transformation and *in vitro* regeneration of the soybean plants. (**A**): The cotyledons were infected by incubating with *A. tumefaciens* carrying the *pBI121::GmDREB6* vector for 30 min; (**B**): Co-cultivation in the dark with CCM for 5 days; (**C**): The cotyledons were cultured in SIM multi shoot regeneration media supplemented with 2 mg/L BAP and 50 mg/L kanamycin for 2 weeks; (**D**): The cotyledons were removed and cultured on SEM supplemented with 0.5 mg/L GA_3_, 0.1 mg/L IAA, and 50 mg/L kanamycin for 2 weeks; (**E**): Initiation of root growth in the regenerated shoots in RM supplemented with 0.1 mg/L IBA for 20 days; (**F**): The rooted plantlets were transferred to pots containing a mixture of rice husk char and sand in the ratio 1: 1.

Four hundred and fifty cotyledonary nodes from the DT84 soybean cultivar were transformed with the *35S-GmDREB6-c-myc* genetic construct using *Agrobacterium tumefaciens-*mediated transformation (Fig. 1). As a control setup, non-transgenic soybean cotyledons were regenerated in an environment without antibiotics, and 8 non-transgenic soybean plants were selected and transferred to pots in the greenhouse.

**Figure 2:**
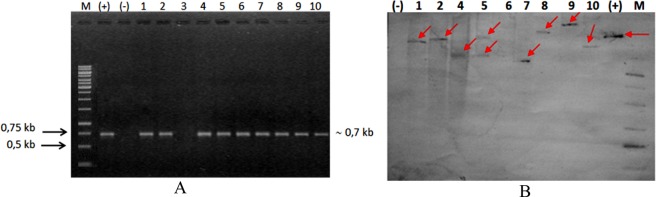
Presence of the *GmDREB6* gene in the GM soybean plants of the T0 generation, evaluated by (**A**): conventional PCR and (**B**): Southern blotting. ↙: The red arrows indicate the presence of *GmDREB6* gene. M: 1.0 kb DNA ladder; (+): *pBI121::GmDREB6* vector; (-): WT: Wild-type; 1–10: The GM soybean plants of the T0 generation were labelled as T0-1, T0-2, T0-3, T0-4, T0-5, T0-6, T0-7, T0-8, T0-9, and T0-10.

The presence of the *GmDREB6* transgene in the transformed soybean plants was confirmed by PCR. The PCR products of the *GmDREB6* transgene from the 10 transgenic soybean plants showed a band that was approximately 0.70 kb, corresponding to the size of the *GmDREB6* transgene (Fig. 2A). Nine transgenic soybean plants in the T0-generation, that showed positive results in the PCR test, were labelled as T0-1, T0-2, T0-4, T0-5, T0-6, T0-7, T0-8, T0-9 and T0-10. Southern blotting was performed for verifying the incorporation of the *GmDREB6* transgene into the genome of these 9 *GmDREB6*-positive soybean plants (Fig. 2B). Results of Southern blotting are depicted in Fig. 2B, which demonstrates that the DNA bands were observed in eight of the nine *GmDREB6*-positive soybean plants, while no such bands were observed for the T0-6 and non-transgenic (control) plants. T0-5 showed 2 DNA bands corresponding to 2 copies of the gene, while the remaining lines, T0-1, T0-2, T0-3, T0-4, T0-7, T0-8, T0-9 and T0-10, showed 1 copy only, indicating that the transformation efficiency was 1.78%.

Among the eight plants in the T0 generation that were successfully transformed, five plants, namely, T0-2, T0-4, T0-7, T0-9 and T0-10 produced flowers as well as seeds in the T1 generation, and were labelled as T1-2, T1-4, T1-7, T1-9 and T1-10. The seeds obtained in the T2 generation were labelled as T2-2, T2-4, T2-7 and T2-10.

### Analysis of the recombinant DREB6 protein

**Figure 3:**
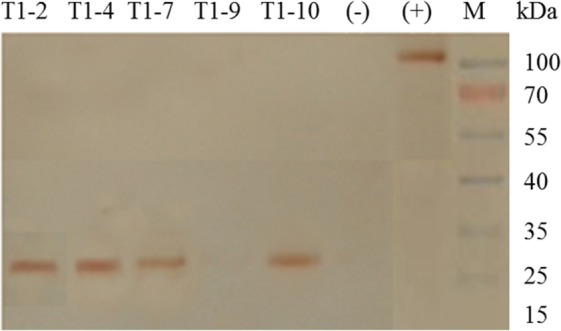
Western blot for the recombinant DREB6 protein in the GM soybean plants of the T1 generation. M: standard protein ladder; T1-2, T1-4, T1-7, T1-9 and T1-10: T1 GM soybean plants; (-): WT; (+): > 100 kDa protein with the c-myc tag.

The recombinant DREB6 protein has the c-myc tag at the C terminal, which is different from the endogenous DREB6 protein, and allows the detection of the recombinant DREB6 protein by western blotting using a c-myc antibody. Leaves from the transgenic lines of the T1 generation were used for analysing the expression of the recombinant DREB6 protein. The results of western blot analysis revealed a band of approximately 27, which corresponded to the molecular weight of the recombinant DREB6 protein kDa, in four GM soybean lines, T1-2, T1-4, T1-7 and T1-10; no band was identified in T1-9 line and non-transgenic soybean plants (Fig. 3). The results of recombinant DREB6 protein expression analysis in GM soybean plants revealed that the *GmDREB6* transgene was inherited from one generation to the next.

### Analysis of the expression of the *GmP5CS* gene in the GM soybean lines and non-transgenic plants

**Figure 4:**
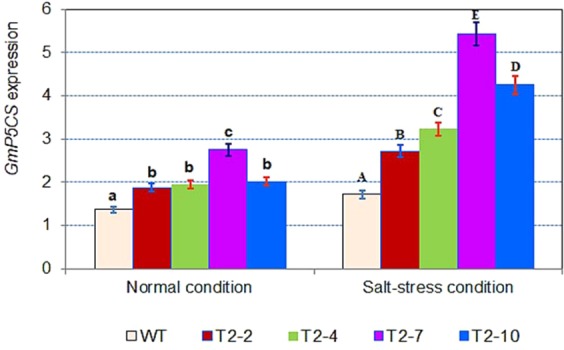
The expression pattern of the *GmP5CS* gene in the four GM lines of the T2 generation and the non-transgenic plants (WT) as determined by qRT-PCR. *Actin* (152 bp) was used as the reference gene. WT: Wild-type; T2-2, T2-4, T2-7 and T2-10: GM soybean lines in T2 generation. The different letters above the columns indicate statistically significant differences (P < 0.05) as measured by Duncan’s tests. The data represents the mean of three biological replicates ± standard error.

Analysis of *GmP5CS* gene expression revealed that the expression was higher in the GM lines than in the non-transgenic plants, under both normal and treated conditions (Fig. 4). The level of *GmP5CS* expression in the transgenic lines ranged from 1.36 to 2.01 folds under normal conditions, and from 1.58 to 3.16 folds under conditions of salt stress. The expression level of the *GmP5CS* gene was highest in the T2-7 line (3.16 folds under conditions of salt stress). Duncan’s test was performed and results revealed that the increase in the expression level of *GmP5CS* gene in the GM soybean lines overexpressing the *GmDREB6* gene under normal and salt treated conditions, was significantly (P < 0.05), and was differentially expressed in the non-transgenic plants and transgenic soybean lines.

### Analysis of proline content in GM and non-transgenic soybean plants under conditions of salt stress

The proline content of the four GM soybean lines, T2-2, T2-4, T2-7 and T2-10 was assayed after the plants were subjected to salt stress for 7 days. The results demonstrated that the proline levels were higher in the GM plants than in the non-transgenic plants. Increase in proline content ranged from 247.83% to 300.00%, compared to that of the plants growing under normal conditions. Among the GM plants, the T2-7 GM line had the highest proline content under conditions of salt stress (P < 0.001) (Table 1). These results suggested that GM lines had a higher salt tolerance than the non-transgenic plants, and that the T2-7 line had the highest salt tolerance among the GM plants studied herein.

**Table 1:** Proline content of the GM soybean lines of the T2 generation and the WT plants after being subjected to salt treatment for seven days.

Non-transgenic plants and GM soybean lines	Proline content of plants under normal conditions (μmol/g)^A^	Change in proline content of GM soybean lines after being subjected to salt stress for 7 days		
		Proline content (μmol/g)^B^	Rate of increase compared to normal condition (%)	Rate of increase compared to non-transgenic plants (%)
WT	1.57^a^ ± 0.027	3.74^a^ ± 0.081	238.22	100.00
T2-2	1.84^b^ ± 0.041	4.56^b^ ± 0.099	247.83	121.93
T2-4	2.32^c^ ± 0.034	6.16^c^ ± 0.044	265.52	164.71
T2-7	2.59^c^ ± 0.029	7.77^d^ ± 0.069	300.00	207.75
T2-10	2.34^c^ ± 0.056	6.29^c^ ± 0.042	268.80	168.18

*Note*: WT: Wild-type; T2-2, T2-4, T2-7, T2-10: GM soybean lines of T2 generation. ^A,B^ Different letters in the same column indicate significant differences as measured by Duncan’s test at P < 0.001. The symbol ± represents the standard error.

## Discussion

As sessile organism, plants need to have a refine system to percept and respond to abiotic environmental conditions in order to survive and reproduce. For important economic crops, such as soybean, keep yield or decrease losses due to abiotic stresses in another task. Usually, physiological and agronomical responses start with molecular switches that triggers responsive-genes expression. Among these genes, DREB (dehydration responsive element binding proteins) are important plant transcription factors (TFs) that bind to specific regions on promoters’ genes activating the expression of mechanisms to cope cellular dehydration^12^, triggering metabolic and physiological responses that help the plant to cope with abiotic stresses, such as drought, heat and salt conditions. It was previously demonstrated that DREB is a trans-acting factor that can bind to the DREB/CRT (C-repeat) sequence, containing an A/GCCGAC motif, for activating the expression of genes involved in the stress signalling pathways of plants. A single 60 amino acid-long DNA binding AP2 domain of DREB/CBF proteins allows them recognize and bind as a single molecule to drought/cold/salt-stress responsive promoter elements. Therefore, DREBs/CBFs have been identified in a wide variety of plants and genetically engineered to produce transgenic generations with higher tolerance to abiotic tresses using different promoters^13^. DREB1 and DREB2, which are highly involved in cold and drought response pathways, are well described in many plants. However, studies on *DREB6* genes are still limited^14^.

In this study, we selected the *GmDREB6* gene, a member of the *DREB* subfamily, found in the soybean genome^15^, for introduction and overexpression in soybean, with the aim of improving salt tolerance in soybean. Enéas *et al*.^19^ identified the DREB gene subfamily in common bean and studied their expression. 54 putative *PvDREB* genes isolated from the common bean genome were defined and divided into six main subgroups. Additionally, four *PvDREB* genes were isolated and analyzed for their responses to dehydration, high salinity, low-temperature, and abscisic acid treatment. Among of which, the expression of *PvDREB1F* and *PvDREB5A* related by drought, salt, cold, and ABA; *PvDREB2A* and *PvDREB6B* were induced under dehydration and cold conditions. The study aided the understanding of the molecular mechanisms associated with drought, salt, and cold tolerance in common bean^16^.

**Figure 5:**
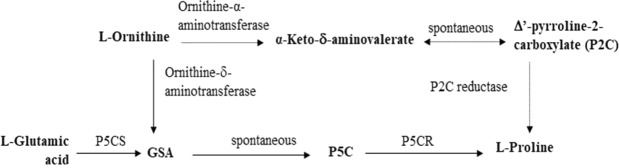
The proline biosynthesis pathways in plants^29^.

Another important response to abiotic stresses is osmotic adjustment which is closely associated with proline content in plant cells. In addition, beside playing a role as an osmolyte, proline also acts as a metal chelator and an antioxidative defence molecule in response to abiotic stresses, especially salt and drought stresses. Stress resistance of plants is strengthened by applying exogenous proline at low concentrations under salt-stressed conditions as well as water deficiency conditions. It is known that the *P5CS* gene, which encodes for proline, plays a critical role in regulating stress response, which induces the accumulation of proline during abiotic stress^17^. Proline accumulation is one of main causes for the increase in osmotic pressure, which in turn enhances the ability of the plants to retain water^8^. The proline biosynthesis pathway in plants involves the participation of two key enzymes, Δ′-pyrroline-5-carboxylate reductase (P5CR) and Δ′-pyrroline-5-carboxylate synthetase (P5CS) (Fig. 5). P5CS is encoded by the *GmP5CS* gene, and the promoter region of the *GmP5CS* gene contains GT-1 region^11^, where *GmDREB6* TF can bind and active gene expression. An increase in proline accumulation, induced by an increase in the expression of the *P5CS* gene, as identified herein in the GM soybean lines, and especially the T2-7 line, could protect the plants against oxidative and osmotic stresses^18^. Previous studies have demonstrated that the overexpression of P5CS increases proline accumulation in transgenic potato plants, common bean, and vegetable soybeans^19–21^. Research of Hmida-Sayari *et al*.^19^ showed that proline content in in transgenic potato plants was higher than in control plants. Noticeably, in transgenic potato plants proline accumulation was enhanced at 100 mM NaCl and salinity tolerance was improved. Consequently, tuber yield and weight were less reduced than non-transgenic plants. Chen *et al*.^19^ studied on the *P5CS* expression in *Vigna aconitifolia* and created genetically modified plants which contain proline content increased 1.5 times compared to control plants after 48 h in salt stress treatment. Zang *et al*. (2015) over-expressed *P5CS* gene in soybeans and obtained T2, T3 transgenic lines which had high salt tolerance and higher proline content than wild-type plants.

Research on the expression patterns of *PvP5CS* in common bean under drought, cold and salt stresses showed that these abiotic stresses lead to increase remarkably the expression of *PvP5CS* in common bean leaves. The results of the transcriptional expression levels of *PvP5CS* in common bean plants after salt treatment were higher than the control up to 16.3-fold at 2 h after treatment. In addition, proline accumulation increased from 1.3-fold at 2 h to 2.7-fold at 6 h treatments^20^.

The results of this study demonstrated that the overexpression of the *GmDREB6* gene increased the transcription levels of the *GmP5CS* gene, which was responsible for the higher intracellular proline content in plants growing under conditions of salt stress (Table 1).

We therefore opine that the overexpression of the *GmDREB6* gene in soybeans enhanced their salt tolerance by increasing the transcriptional expression of the *P5CS* gene that resulted in the accumulation of proline, which plays an important role in stress response.

## Conclusion

The *35S-GmDREB6-c-myc* construct was successfully introduced into soybean by *Agrobacterium*-mediated transformation, producing GM lines up to T2 generation. The expression levels of the *GmDREB6* gene and the accumulation of proline increased in the T2 generation and were higher than those of the wild-type (WT) plants under normal and salt treated conditions. The results of this study demonstrated the effectiveness of the overexpression of the *GmDREB6* gene in increasing proline accumulation in GM soybean plants and suggested that *GmDREB6* could act as a potential candidate for genetic engineering for improving tolerance to salt and drought stresses, due to the crosstalk response mechanisms triggered by these conditions.

## Materials and methods

### Materials

**Figure 6:**
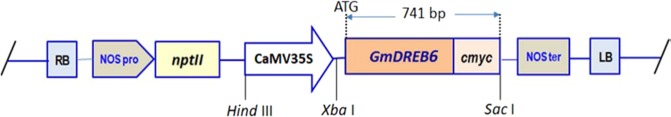
Diagrammatic representation of the *pBI121::GmDREB6* vector used for the *Agrobacterium*-mediated transformation. *LB*: left T-DNA border; *RB:* right T-DNA border; *nptII*: neomycin-phospo-transferase II; CaMV*35S:* cauliflower mosaic virus 35S promoter; *GmDREB6* gene isolated from the mRNA of the soybean plants*; c-myc:* nucleotide sequence encoding the c-myc peptide. Nospro: nopaline synthase promoter; Noster: nopaline synthase terminator; the restriction sites for *Hind III*, *Xba I*, and *Sac I* are indicated by solid black lines.

Seeds from the DT84 soybean cultivar were provided by Agricultural Genetics Institute, Vietnam. Recombinant *A. tumefaciens* CV58 strain carrying the expression vector *pBI121::GmDREB6* was provided by the Department of Genetics and Modern Biology, School of Biology, Thai Nguyen University of Education, Vietnam. The *GmDREB6-c-myc* construct comprises the *GmDREB6* gene, which contains a 693 bp coding region, an 8 bp segment (GCTCTAGA) at the 5′ end containing the cutting site for *XbaI*, and a 7 bp segment (GAGCTCG) at the 3′ end containing the cutting site for *SacI*. A segment of 33 bp, encoding for the c-myc antigen, was added to the 3′ end (Fig. 6). This was used to transform the soybean cotyledon by *Agrobacterium*-mediated transformation. This vector is under the control of the constitutive promoter CaMV 35 S (cauliflower mosaic virus). Two marker genes were also present in the cassette structure: the *nptII* gene (neomycin phospotransferase II), which confers resistance to the antibiotic kanamycin, and was used to select the colonies containing the transgene; and the *c-myc* gene (encoding the c-myc peptide), which functions as an antigen for the detection of the target protein in the Western blot.

### *Agrobacterium*-mediated transformation of soybean

*Agrobacterium*-mediated transformation through the cotyledonary node of soybean and the regeneration of soybean plants were performed according to the methodology previously described by Olhoft *et al*.^22^. Sterilised soybean seeds were germinated on Murashige and Skoog (MS) medium. After 4 days, the cotyledons were used as the materials to be transformed. The cotyledons were submerged into the bacterial suspension for 30 min and then transferred to a non-antibiotic co-culture medium (CCM). The transformed samples were washed for 10 min in a shoot induction medium (SIM) containing 400 mg/L cefotaxime and cultured in the SIM with 50 mg/L kanamycin (1^st^ time). After 2 weeks, the sample was transferred to the SIM with 75 mg/L kanamycin (2^nd^ time). The shoot clusters surviving on the selected media were removed from the cotyledons and transferred to a shoot-growing medium (SEM) containing 50 mg/L kanamycin. When the shoots grew to a length of approximately 3–4 cm, they were transferred to a rooting medium (RM) with 50 mg/L kanamycin for the formation of a complete plant. The healthy plantlets were transferred to pots containing rice husk ash: sand in a ratio of 1:1. After about 1–2 weeks, the surviving plants were transferred to a greenhouse. The soybean plants that regenerated from the transformed cotyledons under *in vitro* conditions were designated as the T0 generation. The seeds from the plants of the T0 generation were considered as the T1 generation, and the seeds obtained from the T1 plants represented the T2 generation.

### Confirmation of the *GmDREB6* transgene in the transgenic plants

The total DNA was isolated from the young leaves of the regenerated soybean plants, according to the method described by Saghai-Maroof *et al*.^23^, and subsequently examined on a 0.8% agarose gel. PCR was performed to confirm the presence of the *GmDREB6* transgene in the regenerated soybean plants. The nucleotide sequences of the PCR primers *SoyDREB6F/SoyDREB6R* are enlisted in Table 2. The PCR was performed using a final volume of 20 μL and contained 0.5 μL of each primer (10 pmol/µL), 12.5 mL 2x Master mix, 2.0 µL cDNA (500 ng/mL), and 4.5 µL water. The PCR, set by the conditions of the thermocycler, comprised the following steps: an initial denaturation at 95 °C for 5 min, followed by 35 cycles of temperature cycling at 95 °C for 20 s, 58 °C for 20 s, and 72 °C for 30 s, and a final elongation at 72 °C for 10 min. The PCR products were excised from a 1.0% agarose gel and purified by a gel extraction kit (Qiagen, Venlo, Netherlands) according to the manufacturer’s instructions.

**Table 2:** The nucleotide sequences of the primer pairs used in the PCR and for synthesis of the DNA probes.

Primers	Nucleotide sequence (5′–3′)	Size
*GmREB6-F/GmDREB6-R*	CATAGAAGAAGCCACTAACACTACA	741 bp
	ATTCAGATCCTCTTCTGAGATGAGT	
*nptII-F/nptII-R*	GAGGCTATTCGGCTATGACTG	963 bp
	ATCGGGAGCGGCGATACCGTA	
*qGmP5CS-F/qGmP5CS-R*	CGAACTGAGCTTGCAGAGGGGC	165 bp
	TCGCTTAGCCTCCTTGCCTCC	
*qAct-F/qAct-R*	CCTAGCATTGTTGGTCGTCCTC	152 bp
	CATATCATCCCAGTTGCTAACAAT	

The DNA of the GM plants that contained the *GmDREB6* transgene, and were therefore successfully amplified by PCR, were subjected to Southern blot analysis^24^. The analysis was performed to detect the presence of the *nptII* gene within the *pBI121::GmDREB6* vector. Genomic DNA samples from the GM soybean plants of the T0 generation that were successfully amplified by PCR were digested overnight using the *SacI* restriction enzyme at 37 °C. After that, the samples were separated on a 1.0% agarose gel and transferred to a cellulose membrane. The *nptII* gene within the *pBI121::GmDREB6* vector was amplified using PCR primers *nptII-F/nptII-R* (Table 2), and the probe was labelled with biotin-11-dUTP using a Biotin DecaLabel DNA Labeling Kit.

### Analysis of the expression of the recombinant DREB6 protein in the GM soybean plants

The total protein was extracted from the leaves of the positively-transformed GM soybean plants of the T1 generation, and subjected to SDS-PAGE using a 10% gel as described by Laemmli^25^. The samples were then transferred to nitrocellulose membranes for Western blot analysis. The membranes were subsequently blocked overnight in blocking solution (5% skim milk in Phosphate Buffered Saline (PBS) - Tween) and incubated with primary antibody (c-myc). The samples were then shaken for 3 h, followed by three washes with PBS (1×), and incubated with a secondary antibody for 2 h. Mouse monoclonal antibody conjugated with c-myc was used as the primary antibody by diluting with 5% milk in PBS at 1:700, while anti-mouse IgG antibody conjugated with horse radish peroxidase and diluted with 5% milk in PBS at 1:4000 was used as the secondary antibody. The results were observed with 3,3′,5,5′-tetramethylbenzidine^26^.

### Salt stress treatment

The seeds of the T2 generation, namely, T2-2, T2-4, T2-7 and T2-10, and those of the WT plants were germinated and grown in a shaded greenhouse at a diurnal temperature of 23 °C and a nocturnal temperature of 20 °C, under a 16 h/8 h- light/dark cycle. At the three-leaf stage (V3), the GM plants of the T2 generation were treated with sodium chloride (NaCl) solutions at different concentrations of 0 (control), 150 and 300 mM for seven days. The plants were watered every 2 days with the 3 different NaCl concentrations. Therefore, the plants in the control setup received plain water without NaCl (0 mM), while the entire experimental plants were watered three times with NaCl solution; the first time was on day 1 with NaCl solution at a concentration of 150 mM, the second and the third times were on day 3 and day 5 respectively, with NaCl solution at a concentration of 300 mM. The leaves of the experimental and control plants were collected on day 7 from the beginning of the experiment, following which they were analysed for determining the transcriptional level of the P5CS gene and the proline content.

### Quantitative reverse transcription polymerase chain reaction (qRT-PCR) analysis

The expression level of the GmP5CS gene in the GM and WT soybean plants, under normal and salt treated conditions, was quantitatively analysed using SYBR Green I fluorescent dye. The total RNA from the leaves of the T2 generation was extracted with the TRIzol kit and cDNA was synthesised using the First Strand cDNA Synthesis Kit, according to the manufacturer’s instructions. The actin gene (SAc1 gene, GenBank accession number: J01298.1) was used as the reference for normalisation. The qRT-PCR test was performed using a final volume of 20 μL, which comprised 0.5 μL of each primer (10 pmol/µL), 12.5 mL 2x Master mix, 2.0 µL cDNA (500 ng/mL), and 4.5 µL water. The qRT-PCR, set by the conditions of the thermocycler, comprised the following steps: an initial denaturation at 95 °C for 10 min, followed by 45 cycles of temperature cycling at 95 °C for 10 s, at 58 °C for 10 s, and at 72 °C for 20 s, following which the flow temperature was analysed. When the flow temperature increased from 65–95 °C for 1 min, the fluorescent signals were collected. The gene expression levels were quantified using the R = 2^−∆∆Ct^ method described by Livak and Schmittgen^27^. The results are presented relative to the results of the genes encoding actin.

### Analysis of the proline content of the transgenic plants

The proline content of the GM and WT soybean plants growing under normal and salt treated conditions were subsequently analysed. The proline content of the GM plants was determined by the method described by Bates *et al*.^28^. The levels of proline in the leaves of the transgenic and non-transgenic plants were analysed after subjecting them to salt stress for seven days. Approximately 0.5 g of the plant material was homogenised in 10 ml of 3% aqueous sulfosalicylic acid and the homogenate was centrifuged at 12000 rpm for 10 min. A mixture comprising 2 mL of the filtrate was made to react with 2 mL of acid ninhydrin and 2 mL of glacial acetic acid in a test tube for 1 h, at 100 °C. The reaction was arrested in an ice bath and the chromophore was extracted with 4 mL toluene, following which its absorbance was determined at 520 nm using a BioMate spectrophotometer. The proline content was expressed as μmoL/g of the fresh mass.

### Statistical analyses

All the data were subjected to one-way analysis of variance using SPSS software. The data were analysed by Duncan’s test (P ≤ 0.05, 0.001).
